# Complete Genome Sequence of *Lacinutrix venerupis* DOK2-8 Isolated from Marine Sediment from the East Sea, Republic of Korea

**DOI:** 10.1128/genomeA.01485-17

**Published:** 2018-01-18

**Authors:** Se Ra Lim, Dae Gwin Jeong, Won-Jae Chi, Ji Hyung Kim

**Affiliations:** a Infectious Disease Research Center, Korea Research Institute of Bioscience and Biotechnology, Daejeon, Republic of Korea; b Biological and Genetic Resources Assessment Division, National Institute of Biological Resources, Incheon, Republic of Korea

## Abstract

*Lacinutrix venerupis* has recently been considered a potential fish pathogen. Here, we report the complete genome sequence of *L. venerupis* DOK2-8, which possesses several virulence-related genes. This strain may be potentially virulent to other marine organisms, and its genomic information will provide important insights into the biodiversity of the genus *Lacinutrix*.

## GENOME ANNOUNCEMENT

The genus *Lacinutrix*, which is a member of the family *Flavobacteriaceae* (*Proteobacteria*: *Gammaproteobacteria*) was created in 2005 and comprises 10 valid marine species, including *L. algicola*, *L. cladophorae*, *L. copepodicola*, *L. gracilariae*, *L. himadriensis*, *L. iliipiscaria*, *L. jangbogonensis*, *L. mariniflava*, *L. undariae*, and *L. venerupis* (1, 2). Although all of these species were considered nonpathogenic (2), *L. venerupis* was recently found to be associated with disease outbreak among marine fish (3). Four draft genomes (*L. algicola*, *L. himadriensis*, *L. jangbogonensis*, and *L. mariniflava*) are currently available in the GenBank database; however, the genome of *L. venerupis* has not yet been sequenced. Since 2015, we have screened several indigenous bacterial strains with advantageous characteristics for biotechnological applications. Here, we present the first complete genome sequence of an *L. venerupis* strain isolated from the Republic of Korea.

The DOK2-8 strain was isolated from marine sediment collected from Dokdo, East Sea, Republic of Korea (37°14′25.9″N 131°52′02.6″E), using the standard dilution plating technique on marine agar 2216 (Difco) followed by incubation at 28°C. The 16S rRNA of the isolate (GenBank accession number MG493235) showed >99.9% similarity to *L. venerupis* Cmf 20.8^T^ (HG970752); hence, it was finally classified as *L. venerupis* DOK2-8. The isolate showed strong extracellular proteolytic activity, as determined with the method of Amoozegar et al. (4). Genomic DNA of DOK2-8 was extracted and sequenced at Macrogen, Inc. (Seoul, Republic of Korea), with a PacBio RS II system (Pacific Biosciences) by constructing a 20-kb SMRTbell template library. The PacBio long-read data (1,055,787,767 bp, 122,792 reads) were assembled *de novo* with the Hierarchical Genome Assembly Process (HGAP) version 3.0 and annotated using the NCBI Prokaryotic Genome Annotation Pipeline (PGAP) (http://www.ncbi.nlm.nih.gov/books/NBK174280).

The final assembled circular chromosome of DOK2-8 comprised 3,192,399 bp, with a 30.6% G+C content (92.5% coding region percentage), and encoded 2,857 genes, 2,811 coding sequences, 6 rRNAs (5S, 16S, and 23S), 36 tRNAs, and 4 noncoding RNAs. Overall genome similarities among DOK2-8 and the other four *Lacinutrix* species strains were assessed using the orthologous average nucleotide identity algorithm (5). The highest genome similarity (80.7%) was with *L. algicola* (GenBank accession number LIQH00000000), and the lowest (75.5%) was with *L. himadriensis* (LIQI00000000).

Potential virulence genes in DOK2-8 were identified by searching the Virulence Factor Database (6, 7); consequently, several putative genes involved in capsule biosynthesis, metalloprotease, and *htpB* chaperonin, which were homologous to those of other Gram-negative species, were detected. Moreover, three putative hemolysin genes were detected in the DOK2-8 genome. These results indicate that *L. venerupis* DOK2-8 may have potential virulence to other marine organisms, similar to other species in the family *Flavobacteriaceae* (2), and information regarding its genome will provide important insights into the biodiversity of the genus *Lacinutrix* in the marine niche. To our knowledge, this is the first study to reveal a complete genome sequence in the genus *Lacinutrix*.

### Accession number(s).

*L. venerupis* DOK2-8 was deposited in the Korean Agricultural Culture Collection (KACC) as KACC 19202. The 16S rRNA and complete genome (chromosome) of *L. venerupis* DOK2-8 have been deposited at DDBJ/ENA/GenBank under the accession numbers MG493235 and CP019352, respectively.
